# Effects of Dietary Vibroactivated Clinoptilolite Supplementation on the Intramammary Microbiological Findings in Dairy Cows

**DOI:** 10.3390/ani10020202

**Published:** 2020-01-25

**Authors:** Dražen Đuričić, Tomislav Sukalić, Franjo Marković, Predrag Kočila, Ivona Žura Žaja, Sven Menčik, Tomislav Dobranić, Miroslav Benić, Marko Samardžija

**Affiliations:** 1 Veterinary Practice Đurđevac, Kolodvorska 2, 48350 Đurđevac, Croatia; djuricic@vet.hr; 2 Croatian Veterinary Institute, Department Križevci, Zakmardijeva 10, 48260 Križevci, Croatia; sukalic.vzk@veinst.hr; 3 Belupo Pharmaceuticals, Vargovićeva 4/3, 48000 Koprivnica, Croatia; franjo.markovic@belupo.hr; 4 Animal Feed Factory d.d., Novakova 11, 40000 Čakovec, Croatia; pkocila@gmail.com; 5 Faculty of Veteririnary Medicine University of Zagreb, Heinzelova 55, 10000 Zagreb, Croatia; izzaja@vef.hr (I.Ž.Ž.); sven.mencik@vef.hr (S.M.); dobranic@vef.hr (T.D.); 6 Croatian Veterinary Institute, Zagreb, Savska Cesta 143, 10000 Zagreb, Croatia; benic@veinst.hr

**Keywords:** antibiogram, clinoptilolite, cow, microbiological finding, udder

## Abstract

**Simple Summary:**

This study aimed to determine the effects of dietary vibroactivated clinoptilolite supplementation on the intramammary microbiological findings in dairy cows, causative agents of intramammary infection, and their sensitivity to antibiotics. The cows (*n* = 78) were randomly divided into two groups: the clinoptilolite (CPL)-treated group that received 100 g of clinoptilolite (CPL) in-feed (*n* = 38) from the seventh month of pregnancy to 75 days after calving and the control group (CON) of untreated cows (*n* = 40). Milk samples were taken from each cow on days 7, 25, 45, and 75 postpartum. Different causative pathogens were isolated in 86 udder quarters (7.07%), in 3.87% environmental microflora, and 89.06% were bacteriologically negative. The most effective antibiotics were cefoperazone and amoxicillin-clavulanic acid, while cloxacillin and tetracycline were the least effective. In the CPL group (14 cows), nine pathogens were isolated in 27 quarters, while in the control (CON) group (24 cows), 13 pathogens in 59 quarters. Cows from the CON group had a 1.96 times higher risk of intramammary infection than cows from the CPL group.

**Abstract:**

The aim of this study was to determine the effects of dietary vibroactivated clinoptilolite supplementation on the intramammary microbiological findings in dairy cows, causative agents of potential intramammary infection, and their sensitivity to antibiotics. Cows (*n* = 78) were randomly divided into two groups: CPL-treated group that received clinoptilolite (CPL) in-feed (*n* = 38), i.e., 50 g natural powdered zeolite CPL, twice daily from the seventh month of pregnancy to 75 days after calving, and the control group (CON) of untreated cows (*n* = 40). Milk samples were taken from each cow on days 7, 25, 45, and 75 postpartum. The following causative pathogens were isolated in 86 udder quarters: *Staphylococcus aureus* in 5.81% of positive samples, *Staphylococcus* spp. 9.32%, coagulase-negative *Staphylococcus* (CNS) 22.09%, *Streptococcus uberis* 13.95%, *Streptococcus agalactiae* 1.16%, *Streptococcus* sp. 3.49%, *Escherichia coli* 8.13%, *Enterococcus* spp. 6.98%, *Corynebacterium* spp. 11.63%, *Pasteurella* sp. 10.47%, *Serratia* spp. 2.33%, and *Arcanobacterium pyogenes*, *Citrobacter* sp., *Prototheca* sp., and yeasts each in 1.16% of samples. Additionally, 3.87% of environmental microflora samples (*n* = 47) and 89.06% of udder samples (*n* = 1083) were bacteriologically negative. The most effective antibiotics were cefoperazone and amoxicillin-clavulanic acid, while cloxacillin and tetracycline were the least effective antibiotics in both groups. In the clinoptilolite supplemented (CPL) group (*n* = 38) of 14 cows, nine causative agents of mastitis were isolated in 27 quarters, while in the control (CON) group (*n* = 40) of 24 cows, 13 causative agents of mastitis were isolated in 59 quarters. Cows from the CON group had a 1.96 times higher risk of intramammary infection than cows from the CPL group during the observation period (odds ratio = 1.96, *p* = 0.0031; 95% CI = 1.2570–3.0770).

## 1. Introduction

Zeolites are natural, hydrated, crystalline aluminosilicates composed of SiO_4_ and AlO_4_ linked together by oxygen atoms into three-dimensional frameworks, like a honeycomb structure with microspores. A negative framework charge caused by the presence of aluminium is balanced by the exact number of cations required to make it neutral. These cations are not part of the zeolite network, and can be replaced by other cations [1]. Zeolites have been used in various technological applications as molecular sieves (for separating and sorting various molecules, for water and air purification, for removal of radioactive contaminants, for harvesting waste energy, for environment protection, etc.) in biotechnology and medicine (for detoxication of animal and human organisms, for improvement of nutrition status and immunity of farm animals, for detection of biomarkers, etc.) [2]. Among the many types of natural zeolites, clinoptilolite (CPL), which incorporates a biologically active nanoporous structure, is the most widespread and scientifically studied [3]. The use of CPL in veterinary medicine has shown that it is capable of acting as a detoxicating (including mycotoxins and heavy metals), antioxidant, haemostatic, anti-diarrheic, growth-promoting, antiviral, antibacterial, and immunostimulating agent [3,4]. Various zeolites have been studied as promising materials for hosting ions with antimicrobial activity. The antimicrobial activity usually involves the slow release of antimicrobial metal ions (such as Cu^2+^, Zn^2+^, or Ag^+^) from the zeolite lattice and the inactivation of some pathogenic agents has been ascribed to metal ion held by aluminosilicate lattice [5,6]. It is assumed that CPL might modulate metabolic, endocrine, and antioxidative status in dairy cows and thus improve their health, fertility, and milk production [7].

Inflammation of the mammary gland and udder tissue (mastitis) is one of the most economically important health issues of dairy cattle [8]. Although the diagnosis of clinical mastitis (CM) is relatively simple, subclinical mastitis (SCM) shows no clinical signs of inflammation. A positive California Mastitis Test (CMT) and increased somatic cell count (SCC) in milk can facilitate diagnosis, as can the isolation and culturing of causative agents from milk samples. Early detection of subclinical mastitis in the dairy industry is of great significance in preventing economic losses [9,10,11]. There are many different microorganisms (mainly bacteria) that have been identified as the causative agents of mastitis. Predominantly in cases of SCM, *Staphylococcus (S.) aureus* is considered a major mastitis pathogen, while the epidemiology of coagulase-negative staphylococci (CNS) is more controversial [12]. *Streptococcus uberis* (*Str. uberis*) is the most frequently isolated bacterium in cases of clinical mastitis, followed by *Escherichia coli* (*E. coli*) and *Streptococcus agalactiae* (*Str. agalactiae*) [13]. There is an increased risk of finding *S. aureus*, *Streptococcus uberis* or *Str. dysgalactiae* in milk samples from chronically infected cows [14]. Intramammary application of antibiotics is the most common form of therapy. The advantage of antibiotic mastitis therapy is the potentially high cure rate with effective selection of the appropriate medicine and prompt treatment. The major disadvantages are potential residues in milk and meat and the development of antimicrobial resistance [15,16,17,18].

The aim of this study was to determine the effects of dietary vibroactivated clinoptilolite supplementation on the intramammary microbiological findings in dairy cows, the causative agents of potential intramammary infection, and their sensibility or resistance to antibiotics.

## 2. Materials and Methods

### 2.1. Animals, Housing, and Feeding

The research protocol and animal management were in compliance with Directive 2010/63/EU of the European Parliament (2010) on the protection of animals used for scientific purposes and of the Council on the protection of animals used for scientific purposes. The experimental animals and their operating procedures in this study were in compliance with the regulations of national and local animal welfare agencies, and were approved by the Ethics Committee of the Faculty of Veterinary Medicine, University of Zagreb, Croatia (records No.: 640-01/14-17/51; file No.: 251-61-01/139-14-1) and Veterinary and Food Safety Directorate, Ministry of Agriculture, Republic of Croatia (records No.: UP/I-322-01/14-01/111; file No.: 525-10/0255-15-2).

The study was carried out using 78 clinically healthy Holstein Friesian breed cows between 2 and 4 years of age, held at a private dairy farm (Pleško family farm) near Đurđevac, Koprivnica-Križevci County, Croatia (coordinates 45°59′ N, 17°03′ E). All cows were housed in a free-stall barn with straw bedding. Animals were randomly divided into two groups: CPL-treated group that received clinoptilolite (CPL) in-feed (*n* = 38), i.e., 50 g natural powdered zeolite CPL modified by vibroactivation and micronisation (Vibrosorb^®^, Viridisfarm, Podpićan, Croatia), twice daily from the seventh month of pregnancy to the 75 days after calving, and a group of untreated animals (CON group) (*n* = 40). All cows suffering from clinical mastitis, metritis, lameness, milk fever, abomasal displacement, retained placenta, or cystic ovarian dysfunction were excluded from the study. Daily diet consisted of 18 kg grass silage, 8 kg corn silage, 3 kg meadow hay, and 5 kg concentrate for dairy cows (1 kg contained: 19% crude proteins; 13.5% moisture; 10% crude fibres; 9% ash; 1% Ca; 0.7% P; 0.25% Na; 10,000 I.U. vitamin A; 1500 I.U. vitamin D_3_; 20 mg vitamin E; 40 mg Mg; 5 mg Cu; 20 mg Fe; 20 mg Zn; 20 mg Mn; 0.6 mg I; 0.05 mg Co; 0.1 mg Se). Cows were fed a 50:50 forage-to-concentrate ratio during lactation on a DM basis and were milked twice daily at 6 a.m. and 4 p.m. Before calving, during the drying-off period, the dietary forage-to-concentrate ratio was 75:25 on a DM basis. After calving, during early lactation, the forage-to concentrate ratio on a DM basis was 60:40. Water was available ad libitum.

### 2.2. Milk Sampling and Analytical Procedures

Milk samples were taken from all of four udder quarters separately and as a composite sample of milk from each cow for determination of SCC on days 7, 25, 45, and 75 postpartum. After washing and drying, teat ends were disinfected with cotton swabs soaked in 70% ethanol. The first few streams were discarded, and samples from each udder quarter were collected in sterile tubes for microbiological examination. Samples were cooled and immediately transferred to the laboratory at the Croatian Veterinary Institute, Zagreb, and analysed. The methods of isolation and identification of microorganisms were carried out according to the methods recommended by the National Mastitis Council [19]. The milk samples were inoculated onto nutrient agar (5% ovine blood and 0.1% aesculin) and incubated at 37 °C. Grown colonies were stained according to Gramm (Merck, Darmstadt, Germany), checked for catalase and oxidase production, and further subcultured onto differential or selective media (Baird-Parker agar (staphylococci), coagulase production (media with 0.5 mL rabbit plasma), CAMP test (Streptococcus agalactiae), Gram-negative bacteria (MacConkey agar and Triple Sugar Iron agar (Merck)). Causative pathogens were finally identified by biochemical profiling using Micronaut identification systems (Merlin Diagnostika, Bornheim, Germany) for Gram-positive bacteria and Gram-negative fermentative bacteria. For yeasts and moulds were used Sabouraoud agar.

Commercial discs with the following antibiotics and chemotherapeutics were used to perform the antibiogram: amoxicillin and clavulanic acid, ampicillin, cefoperazone, enrofloxacin, kanamycin, cloxacillin, lincomycin, neomycin, novobiocin, penicillin, streptomycin, sulphamethoxazol-trimethoprim, and tetracycline (according to performance standards for antimicrobial disc and dilution susceptibility tests for bacteria isolated from animals; approved standard (third edition)).

### 2.3. Clinoptilolite Specification

Natural clinoptilolite modified by vibroactivation and micronisation (mironised sodium potassium magnesium calcium aluminosilicate hydrate) containing particles reduced to the size of 4.22 μm (D75) via Vibrosorb^®^ was procured from Viridisfarm, Podpićan, Croatia. Its chemical composition is SiO_2_ 62–68%, Al_2_O_3_ 9–13%, CaO 2.5–6%, MgO 0.25–1.0%, K_2_O 0.5–1.0%, Na_2_O 0.5–2.0%, and Fe_2_O_3_ 0.2–1.5%. Density at 20 °C is 2.0–2.4 g/cm^3^. The microbial mutagenicity assay (Ames test) was negative.

### 2.4. Statistical Analysis

The data were analysed using the program package Stata 13.1 (Stata Corp., Lakeway Drive, College Station, TX, USA). The values obtained for SCC were log-transformed prior to statistical analysis to normalise the distribution. The number of infected udder quarters was compared between the groups of cows by using Fisher’s exact test. Probability for intramammary infection was calculated using logistic regression (odds ratio (OR)).

## 3. Results

**Figure 1:**
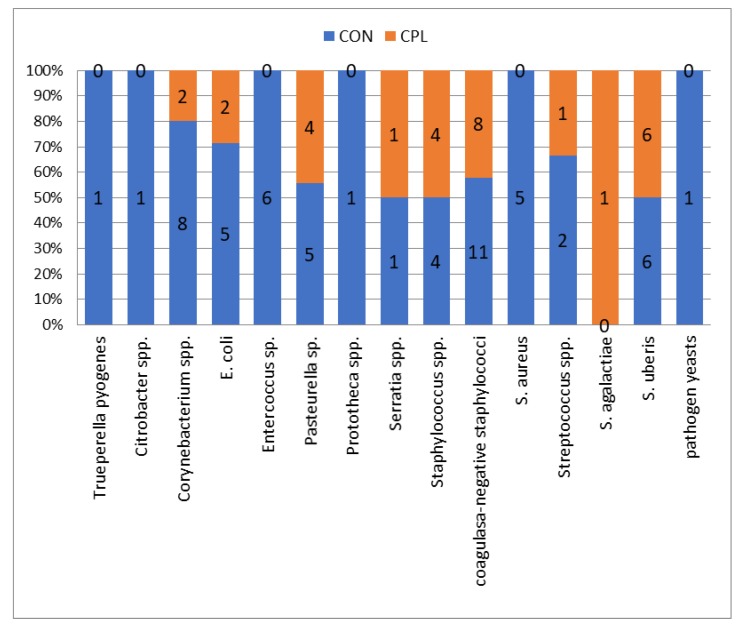
Number and percentage of causative pathogens isolated in the clinoptilolite (CPL) supplemented group (udder quarters *n* = 29) and in not treated group (control (CON)) (udder quarters = 57).

A total of 1216 milk samples were taken from 78 cows, i.e., 304 udder quarters (as eight quarters of seven cows were non-functional). After microbiological examination, 7.07% of milk samples (*n* = 86) gave positive results. The following causative pathogens were isolated in 86 quarters: *Staphylococcus aureus* in 5.81% of positive samples, *Staphylococcus* spp. 9.32%, coagulase-negative *Staphylococcus* (CNS) 22.09%, *Streptococcus uberis* 13.95%, *Streptococcus agalactiae* 1.16%, *Streptococcus* sp. 3.49%, *E. coli* 8.13%, *Entercoccus* spp. 6.98%, *Corynebacterium* spp. 11.63%, *Pasteurella* sp. 10.47%, *Serratia* spp. 2.33%, and *Trueperella pyogenes*, *Citrobacter* sp., *Prototheca* sp., and pathogen yeasts were each isolated in 1.16% of samples (Figure 1). In 3.87% of environmental microflora (*n* = 47) and 89.06% of samples (*n* = 1083), results were bacteriologically negative. The SCC in milk from the CON group of cows did not differ to the CPL group in any of the sampling points (225,925 vs. 202,931, respectively). Recorded differences of SCC between single samplings did not differ significantly in either the CON or CPL group of cows.

A statistically significant difference in the number of infected udder quarters between CPL and CON group of cows was established (*p* < 0.05). During the experiment, 13 causative agents of mastitis were isolated from 59 quarters in 24 cows in the control group. In the CPL group, nine causative agents of mastitis were isolated from 27 quarters in 14 cows. No significant differences in milk yield were found between the two groups of cows. The distribution of the type of causative agents was significantly different between group (*p* = 0.04) (Figure 1). In the CPL groups, the most frequent bacteria were coagulase-negative staphylococci (11/57) as in the CON group (8/29) (see Figure 1). In the current study, cows from the CON group had a 1.96 times higher risk of intramammary infection than cows from the CPL group (odds ratio = 1.96, *p* = 0.0031; 95% CI = 1.2570–3.0770). The most effective antibiotics were cefoperazone and amoxicillin-clavulanic acid, while cloxacillin and tetracycline were the least effective antibiotics in both groups (Table 1). In the front quarters, 60.47% of causative pathogens were isolated and 39.53% in the rear quarters (Table 2).

## 4. Discussion

In the literature [13,14,17], the most commonly isolated bacteria in bacteriological positive samples in clinical mastitis case from middle- and large-scale dairy farm is *S. aureus* (20–40%) followed by the coagulase-negative staphylococci (CNS) (15–30%). However, on a middle-scale family farm, in our study, CNS was the most isolated bacteria in both the CON and CPL groups (19.3% for CPL and 27.58%, for CON, respectively), while *Staphylococcus* spp. and *S. aureus* were below the average. Although, in our study, the incidence of *Streptococcus uberis* was very high in the CPL group (20.69%), followed by *Pasteurella* sp. (13.79%) and *E. coli* and *Corynebacterium* spp. (each 6.9%), in the CON group, there was a surprisingly high incidence of purulent bacteria (*Corynebacterium* spp., 14.04% or *Trueperella pyogenes* (1.16%) in one sample) and bacteria from faecal contamination (*Enterococcus* spp., 10.54% and *E. coli*, 8.77%), which countered findings in Sweden [14] and in Estonia [13]. Similar to our study, a frequent occurrence of enterococci (21.3%) in cow’s milk was determined. Considering the antimicrobial resistance [20], the highest level of resistance was observed to lincomycin, tetracycline, erythromycin, kanamycin, and streptomycin [19], similar to our antibiogram, and in addition to novobiocin, cloxacillin, and enrofloxacin (two samples were moderately resistant). All isolated bacteria were sensitive to amoxicillin-clavulanic acid and ampicillin, while sulphamethoxazole-trimethoprim, cefoperazone, and neomycin were each effective in two samples (numbers seven and nine). Despite the fact that *S. aureus* was the most frequently isolated pathogen, its resistance to antimicrobials was rare (4% of *S. aureus* isolates and 35% of the CNS isolates) [14]. Unlike previous findings, resistance to penicillin was found in *S. aureus* and [15] CNS isolates (61.4% and 38.5%, respectively) [13], which was similar to our study (61.11% and 46.15%, respectively). The most effective antimicrobial substances for the treatment of streptococcal mastitis were β-lactams and macrolides [13,21,22,23,24], as confirmed in our study. The rate of multiresistance to antibiotics was higher in *Str. uberis* (15%) than for other streptococcal species, though in this study it was 12.50% in the CPL group and 26.67% in the CON group. Bacterial resistance to tetracycline was detected the most frequently [24,25,26,27]. Although β-Lactams are the best choice in the treatment of streptococcal mastitis [22,23,24,25,26,28,29,30], in our study, all isolates were fully susceptible to cefoperazone [29], penicillin, and ampicillin. The administration of clinoptilolite alone, and particularly in combination with selenium, increased the antibody titres against *E. coli* in the blood serum of heifers and calves [31]. Clinoptilolite is used as a feed ingredient due to its beneficial properties as an immunostimulant. The indirect action of clinoptilolite on the immune system is also achieved by its antioxidant capacity [32,33]. In this study was established a statistically significant difference in the number of infected udder quarters between the CPL and CON groups of cows. In fact, in the control group, 13 causative agents of mastitis were isolated in 59 quarters (of 24 cows), while in the CPL group, nine causative agents were isolated in 27 quarters (14 cows). The most effective antibiotics were cefoperazone and amoxicillin-clavulanic acid, while cloxacillin and tetracycline were the least effective antibiotics in both groups.

## 5. Conclusions

This study evaluated the effect of dietary vibroactivated and micronised clinoptilolite on the intramammary microbiological findings in dairy cows, causative agents of potential intramammary infection, and their sensitivity or resistance to antibiotics. In the current study, cows from the CON group had a 1.96 times higher risk of intramammary infection than cows from the CPL group during the study period. Therefore, antibacterial, detoxifying, antioxidative, and immunostimulating effects of the administration of the natural, vibroactivated, and micronised zeolite clinoptilolite, two months before calving until 75 days after calving, reduce the incidence of intramammary infections.

## Figures and Tables

**Table 1:** Sensitive, resistant, or moderately resistant bacteria (isolated from udder quarters) to antibiotic action (from a total of 86 positive samples) in the control (CON) and clinoptilolite (CPL) supplemented group of dairy cows.

Group	CON (*n* = 57)		CPL (*n* = 29)		Both Groups			
Sensitivity to Antibiotics	Sensitive				Sensitive		Resistant	Moderately Resistant
antibiotics	*n*	%	*n*	%	*n*	%	*n*	*n*
ampicilin	45	78.95	24	82.76	69	80.23	17	0
amoxicillin-klavulanic acid	48	84.21	23	79.31	71	82.56	15	0
cefoperazone	50	87.72	28	96.55	78	90.70	4	4
enrofloxacin	25	43.86	16	55.17	41	47.67	39	6
kanamycin	22	38.60	8	27.59	30	34.88	51	5
cloxacilin	10	17.54	4	13.79	14	16.27	72	0
linkomycin	20	35.09	14	48.28	34	40.70	52	0
neomycin	26	45.61	9	31.03	35	40.70	51	0
novobiocin	19	33.33	12	41.38	31	36.05	55	0
penicillin	30	52.63	21	72.41	51	59.30	28	7
streptomycin	14	24.56	4	13.79	18	20.93	64	4
sulphametoxazole-trimethoprim	35	61.40	23	79.31	58	67.44	26	2
tetracycline	13	22.81	3	10.34	16	18.60	70	0

**Table 2:** Number of total microbiological positive udder quarters.

Quarter	Front Left	Rear Left	Rear Right	Front Right
quarter tag	1	2	3	4
*n* (positive)	27	18	16	25
%	31.40	20.93	18.60	29.07
total front (1 and 4)	*n* = 52		60.47%	
total rear (2 and 3)	*n* = 34		39.53%	
